# Precision Magnetic Field Sensing with Dual Multi-Wave Atom Interferometer

**DOI:** 10.3390/s23010173

**Published:** 2022-12-24

**Authors:** Wenhua Yan, Xudong Ren, Minkang Zhou, Zhongkun Hu

**Affiliations:** MOE Key Laboratory of Fundamental Physical Quantities Measurements, Hubei Key Laboratory of Gravitation and Quantum Physics, PGMF and School of Physics, Huazhong University of Science and Technology, Wuhan 430074, China

**Keywords:** atom interferometer, magnetometer, optical detection

## Abstract

Precision magnetic field measurement is widely used for practical applications, fundamental research, and medical purposes, etc. We propose a novel quantum magnetometer based on atoms’ multi-wave (3-wave and 5-wave) Ramsey interference. Our design features high phase sensitivity and can be applied to in situ measurements of the magnetic field inside vacuum chambers. The final state detection is designed to be achieved by Raman’s two-photon transition. The analytical solution for applicable interference fringe is presented. Fringe contrast decay due to atom temperature and magnetic field gradient is simulated to estimate reasonable experimental conditions. Sensitivity functions for phase noise and magnetic field noise in a multi-wave system are derived to estimate the noise level required to reach the expected resolution. The validity of the model, dual-channel features on bias estimation, and the quasi-non-destructive detection feature are discussed.

## 1. Introduction

Magnetic field sensing is widely used in resource exploration [1,2], archaeology [3,4], and the medical domain [5,6,7,8,9], etc. In addition to the industrial application, measuring the magnetic field plays a key role in fundamental research [10], aerospace [11,12,13], and geophysics [14], etc. In metrology, countless experiments are related to the Zeeman effect. For example, it is important to estimate the magnetic-field-induced systematic uncertainty in atom clocks [15] and atom interferometers [16,17,18].

The required resolution for resource exploration or geophysical survey is at least a nanotesla [1]. Amplitudes of various biomagnetism signals go much further. For example, magnetoencephalography [19] and magnetocardiography [20] require resolution on a scale of femtoteslas. Optically pumped atomic magnetometers [21,22] and SQUID [23] have proven their sensitivity to femtotesla$/\sqrt{\mathrm{Hz}}$ and even sub-femtotesla$/\sqrt{\mathrm{Hz}}$. Among these state-of-the-art magnetometers, atom magnetometers [6,7,8,9,12,13,20,24,25,26] have been developed for decades. Thermal atom sources lack spatial resolution and control of motion and have a long coherence time. Cold atom sources [27,28,29,30] are expected to make up for these shortages.

Several research groups have realized magnetic field measurement using ultra-cold atomic ensembles. Vengalattore et al. [30] reached a highly sensitive result of magnetic field mapping converted from field-dependent spatial amplitude distribution. Eto et al. [31] measured Larmor frequencies from interferometric fringes and observed the variance of spin to extract information about the magnetic field. Muessel et al. [32] demonstrated quantum-enhanced magnetometry with spin squeezed states in an effective two-level system, leading to a resolution ∼20% better than the standard quantum limit. These magnificent works demonstrated results pointing to the cutting edge of atom magnetometry.

An alternative way to increase measurement sensitivity is to take advantage of the increased fringe slopes in a multi-wave interferometer. Multi-wave interferometers [29,30,33,34,35,36,37] feature higher fringe slopes [38], leading to higher sensitivity and multiple observation ports containing more rich information. There have been some experimental results [39,40] on measuring magnetic fields with Raman two-photon interferometric methods in a two-level system. Experiments [29,30,35,41] have presented interference employing one-photon direct coupling or Raman two-photon coupling [42] in a multilevel system. We focus on the study of magnetometry in a multilevel system by RF coupling.

Here we combine the methodology of atom interferometry, especially the interferometer phase analysis [43], with atom magnetometry. Furthermore, we make use of all spin states on the hyperfine levels. As an example of the ground states in $5^{2}S_{1/2}$ of ${}^{87}\mathrm{Rb}$, by preparing the initial state with atoms in both F=1 and F=2 states, a dual multi-wave interferometer is proposed. The atom source, the initial state preparation and the final state detection in the proposed scheme can be achieved optically. Our design can be applied to the in situ measurement of the magnetic field inside a vacuum chamber.

Our paper is organized as follows. First, we present the modeling in Section 2. Second, in Section 3, phase behavior applicable to magnetic field measurements and the expected resolution are presented. Third, in Section 4, reasonable experimental conditions are presented. Fourth, in Section 5, sensitivity functions to phase noise and magnetic field noise are demonstrated, deducing the required noise level for precision measurement. In the end, discussions about the relationship to the existing atom magnetometers, features for dual-channel magnetometers, multi-state detection and conclusions are presented.

## 2. Modeling

**Figure 1:**
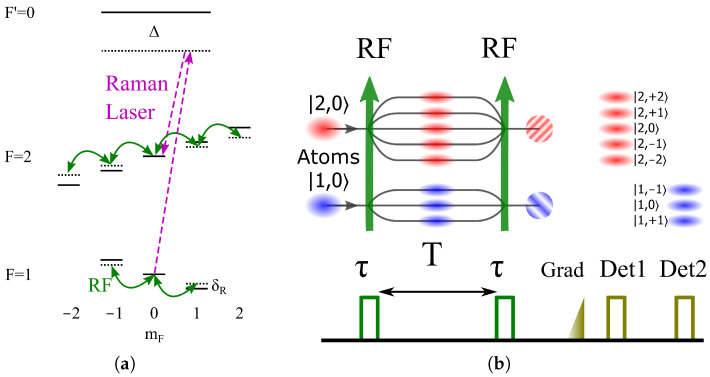
The level scheme (**a**) and the time sequence (**b**) of a dual multi-wave atom magnetometer. (**a**) RF radiation couples different spin states. Raman transition couples $\Delta F=+1,\;\Delta m_{F}=0$ states. For clarity, only $m_{F}=0$ state is sketched. (**b**) Two RF pulses of duration τ to couple spin levels and free evolution time *T* form Ramsey interference in the presence of a magnetic field B. After a pulse of the magnetic field gradient, the fringes of F=2 are read at detection step 1, and those of F=1 are then read at detection step 2.

The level scheme and the sequence of the dual atom magnetometer are depicted in Figure 1. The sequence starts with a cold atom ensemble containing |F=1〉 and |F=2〉 states. This can be achieved by adjusting the repumper (for the $\left|F=1\rangle \to \right|F^{\prime }=2\rangle$ transition) parameter as the intensity or duration, or in a coherent way by employing microwave or Raman transition between |F=1〉 and |F=2〉 states. The fraction of atoms in F=1 and F=2 systems form the two-atom interference magnetometers (AIM). We use the RF signal to perform Ramsey interference [44] between spin levels and then realize spatial separation by a magnetic field gradient pulse. The atoms of F=2 are detected first by resonant absorption imaging. After that, assisted by a microwave or Raman [45] pulse to probe atoms in one specific $m_{F}$ state, the interference fringe in the F=1 system is detected. Similar to the Ramsey interference in a two-wave system, in a multi-wave system, in our case, the relative phase shift between the RF signal and the magnetic-field-induced phase during evolution time *T* pushes atoms to one or the other levels, producing the fringes that can be read by the second RF pulse.

**Figure 2:**
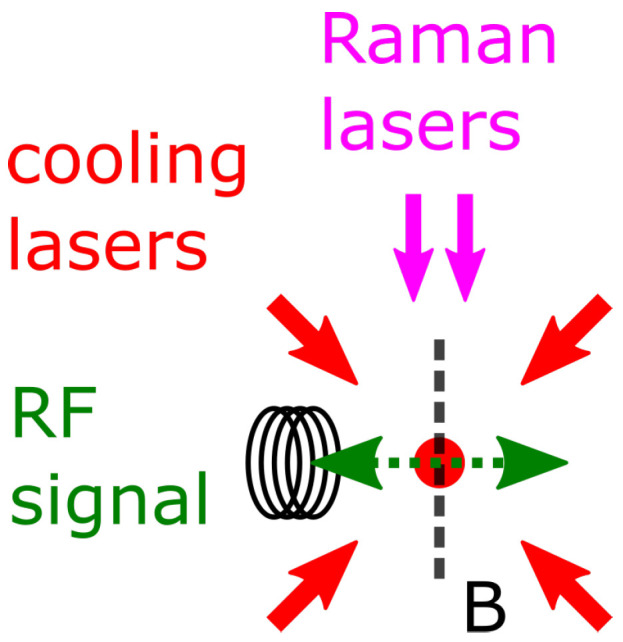
Conceptual setup. Cooling lasers and Raman lasers are pointing to the atom cloud. At the position of the atom cloud, the RF oscillating signal is perpendicular to the bias magnetic field B. Raman lasers are parallel to the B field.

The conceptual design of the proposed scheme is sketched in Figure 2. The facilities of atom cooling lasers and Raman lasers are reported in Ref. [46]. Atom clouds are freely falling during the sequence. Raman lasers can be used for spin-specific detection in the F=1 system in detection step 2 in Figure 1.

Similar to the theoretical framework of Refs. [47,48], when atoms are subjected to the homogeneous magnetic field B, considering the first-order Zeeman effect, the Zeeman levels differ μ·B between each other, where μ is the magnetic dipole moment. We use an oscillating RF signal with the form $B_{\mathrm{RF}}=B_{0}e_{\mathrm{RF}}\cos\left(\omega_{\mathrm{RF}}t+\varphi_{\mathrm{RF}}\right)$ to couple the spin levels, where $B_{0}$ is the RF wave amplitude, $\omega_{\mathrm{RF}}$ is the RF circular frequency, $\varphi_{\mathrm{RF}}$ is the RF wave initial phase and $e_{\mathrm{RF}}$ indicates the direction. The direction of B is taken as the quantization axis. $B_{\mathrm{RF}}$ is linearly polarized and $e_{\mathrm{RF}}$ is perpendicular to B. The RF phase $\varphi_{\mathrm{RF}}$ enables the later phase noise analysis.

Different from Ref. [35], we build the interferometer by using atoms in the $m_{F}=0$ state. Different from Ref. [29], the proposed scheme provides an option to use atoms in both *F* systems. The calculation in Appendix A demonstrates the reason for choosing the $m_{F}=0$ state as the initial state.

## 3. Multi-Wave Ramsey Interference

A typical Ramsey sequence consists of one excitation pulse, a period of free evolution and a final readout pulse for interference fringe. The excitation pulse and the readout pulse are described by $U_{R}\left(\tau \right)$, as in Equations (A4) and (A5). The transfer matrices $U_{\mathrm{Rf}}\left(t\right)$ describing the free evolution are diagonal, with matrix elements $e^{-i\delta t}$, where δ corresponds to the diagonal elements of Equations (A2) and (A3). The matrix forms are Equation (A11) for F=1 and Equation (A12) for F=2. The interferometer phase $\phi_{R}$, indicating the relative phase shift between the RF signal and the magnetic-field-induced phase during free evolution time *T*, is expressed as

$$\phi_{R}=\delta_{R}T=\left(\omega_{\mathrm{RF}}-\kappa B\right)T.$$

The output of the Ramsey interferometer is

(1) $$c\left(\tau +T+\tau \right)=U_{R}\left(\tau \right)U_{\mathrm{Rf}}\left(T\right)U_{R}\left(\tau \right)c\left(0\right).$$

**Figure 3:**
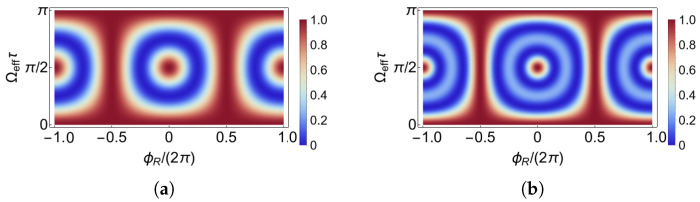
Ramsey fringes observed on |F,0〉 state setting parameters as pulse area $\Omega_{\mathrm{eff}}\tau$ and interferometer phase $\phi_{R}$ in the (**a**) F=1 system and in the (**b**) F=2 system. Colorbars indicate the probability.

First of all, we calculate the center state interferometer fringes as a function of pulse area $\Omega_{\mathrm{eff}}\tau$ and interferometer phase $\phi_{R}$. From Figure 3, for pulse area close to π/4, during $\phi_{R}$ of 2π, the fringes evolve with full contrast by one period. Only for pulse area close to π/2, the fringe evolution periodicity is doubled, leading to higher fringe slopes. Within expectation, the fringes in the F=2 system are sharper than those in the F=1 system, as compared to the width of varying colors in Figure 3a,b. We choose the typical experimental parameters as pulse area of π/4 and π/2, which are more like π/2 pulse and π pulse in a two-level system, for the next step of the analysis.

**Figure 4:**
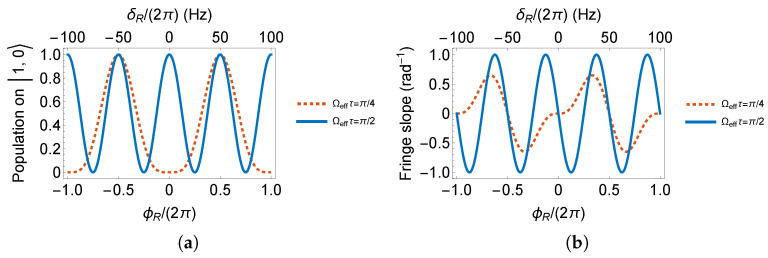
(**a**) Ramsey fringes and (**b**) its slopes on |1,0〉.

**Figure A1:**
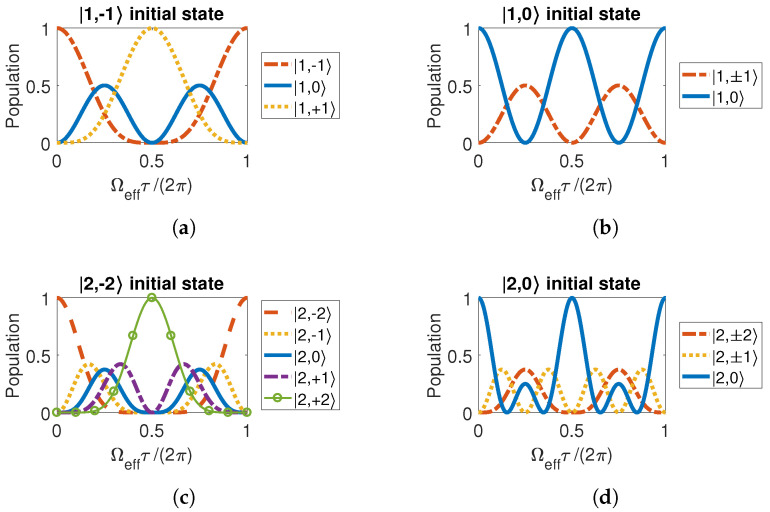
Rabi oscillations in the F=1 system when the initial state is a (**a**) side state and a (**b**) center state. Rabi oscillations in the F=2 system when the initial state is a (**c**) side state and a (**d**) center state.

In the F=1 system, according to Figure A1b, a π/2 pulse spreads all atoms to |1,±1〉 states. Observation on |1,0〉 output will present a zero population. If we set the pulse area to π/4, a minimum as a dark fringe is expected at the Ramsey fringe center, where the interferometer phase is zero. On the contrary, in Figure A1b, a π pulse recovers the full population on |1,0〉. If we set the RF pulse area to π/2, a maximum as a bright fringe is expected at the Ramsey fringe center. The interferometer phase behavior on |1,0〉 is expressed in Equations (2) and (3) and plotted in Figure 4. The evolution time *T* is set to 10ms for demonstration.

(2) $$P_{R1(\pi /4)}=\frac{1}{4}{\left(1-\cos\phi_{R}\right)}^{2}$$

(3) $$P_{R1(\pi /2)}=\frac{1}{2}\left(1+\cos\left(2\phi_{R}\right)\right)$$

From Figure 4, we can see that the phase of a Ramsey interferometer with two π/2 pulses evolves twice as fast as the one with two π/4 pulses.

**Figure 5:**
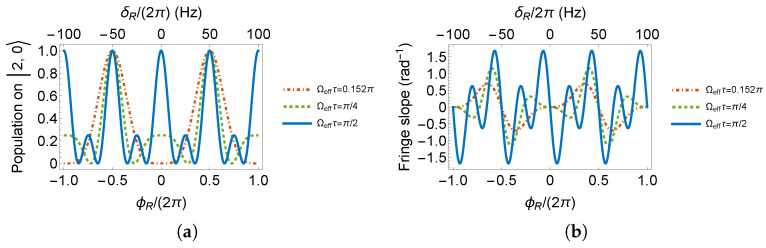
(**a**) Ramsey fringes and (**b**) its slopes on |2,0〉.

In the case of F=2, same as in F=1 system, two π/2 pulses produce a bright fringe center. Different to the case in F=1, the fringe center with two π/4 pulses is no longer a zero population point. According to Figure A1d and the |2,0〉 component in Equation (A10), the full population inversion occurs when $\Omega_{\mathrm{eff}}\tau =1/2\arccos\left(-1/3\right)$. Therefore, we set $\Omega_{\mathrm{eff}}\tau$ as 1/4arccos(−1/3), noted as 0.152π, to produce a dark fringe center. Phase behavior of pulse area of 0.152π, π/4 and π/2 are listed in Equations (4)–(6) and plotted in Figure 5.

(4) $$P_{R2(0.152\pi )}=\frac{1}{16}\left(\sqrt{3}-\left(2-\sqrt{3}\right)\cos\phi_{R}\right){}^{2}\left(1-\cos\phi_{R}\right){}^{2}$$

(5) $$P_{R2(\pi /4)}=\frac{1}{64}\left(3\left(1-\cos\phi_{R}\right){}^{2}-4\right){}^{2}$$

(6) $$P_{R2(\pi /2)}=\frac{1}{16}\left(1+3\cos\left(2\phi_{R}\right)\right){}^{2}$$

The fringe slopes in Figure 4b and Figure 5b are, respectively, 0.66, 1.00, 0.70, 1.17, $1.67\;{\mathrm{rad}}^{-1}$. We conclude that if ${}^{87}\mathrm{Rb}\;5^{2}S_{1/2}$ is chosen to measure the magnetic field by the RF Ramsey method, conditions such as the initial state prepared on |2,0〉, observation on |2,0〉 and pulse area of π/2 yield the highest fringe slope, resulting in the most sensitive measurement.

**Figure 6:**
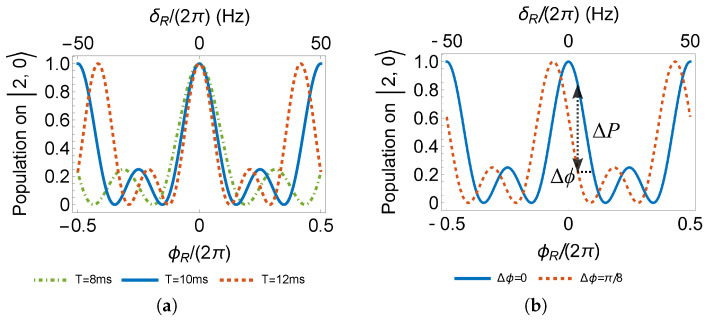
(**a**) Determination of fringe center with different evolution time *T*. (**b**) Demonstration of fringe slope linearity to interferometer phase shift.

The fringes are periodic with the interferometer phase $\phi_{R}$. To determine the fringe center, where $\phi_{R}=0$, one can vary the free evolution time *T* in the Ramsey sequence. The fringe center is overlapped independent of evolution time *T* in Figure 6a. Although the fringes in a multi-wave interferometer are complicated, near the regime of the highest fringe slope, the change in the readout probability is linear to the change in the interferometer phase, as demonstrated in Figure 6b.

**Figure 7:**
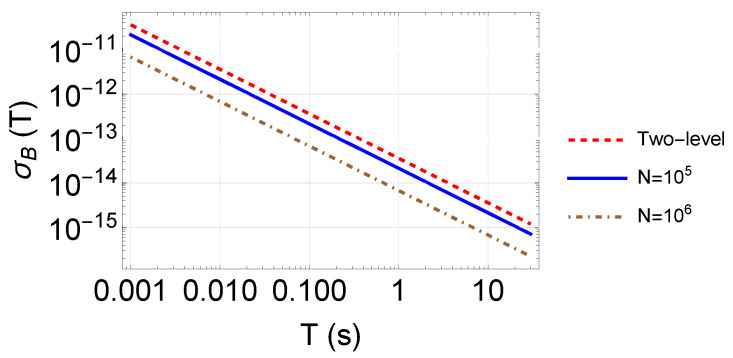
One-shot resolution to magnetic field measurement. The pulse area is π/2, and Equation (6) is chosen as the detection port. The red dashed line as the comparison basis is the resolution employing a microwave in a two-level system.

The resolution limit of magnetic field measurement $\sigma_{B}$ is

(7) $$\sigma_{B}=\frac{\frac{\partial }{\partial \phi }B}{\frac{\partial }{\partial \phi }P}\times \sigma_{P}=\frac{2\hbar /(\mu_{B}T)}{\mathrm{fringe}\;\mathrm{slope}}\times \sqrt{\frac{P(1-P)}{N_{\mathrm{tot}}}},$$

where $\sigma_{P}$ is the uncertainty limited by quantum projection noise [49] and *P* is the probability of detecting atoms at certain interferometer output, i.e., the phase behavior. Taking the calculated fringe slope of Equation (6), we can see from Figure 7 that preparing the initial state on |2,0〉 with $1\times 10^{5}$ atoms, employing two π/2 pulses, and a free evolution time of 21ms is enough to reach a one-shot resolution of 1.0pT magnetic field. Under the same condition, using the microwave to measure the Zeeman shift between |1,1〉 and |2,1〉 results in one shot resolution of 1.7pT, as the dashed line in Figure 7. Despite the advantage in measurement resolution, atom species besides ${}^{87}\mathrm{Rb}$ with a hyperfine ground state of more than five Zeeman levels can produce a fringe slope higher than the demonstration in this paper, leading to better resolution. With optimal conditions such as an atom number of $1\times 10^{6}$, in principle, 6.7s for free evolution is expected to attain the resolution limit of 1.0fT per shot. In the free-falling configuration, the free evolution time of 6.7s requires a path of more than 200m! Rather than increasing the evolution time, increasing the atom number would be the proper strategy. Otherwise, the long evolution time limits the application to microgravity. In addition, the trapped configuration in Section 6.3 also provides a method for a long evolution time.

The noise floor limited by the quantum projection noise reaches the level of state-of-the-art magnetometers. The required resolution for resource exploration or geophysical survey is at least a nanotesla [1]. Amplitudes of various biomagnetism signals go much further. For example, magnetocardiography [6,20] and magnetoencephalography [19] require resolution on a scale of picoteslas or even femtoteslas. To reach the one-shot resolution of 1pT, reasonable experimental conditions and noise analysis before the experiment should be considered.

## 4. Experimental Condition

In addition to the atom number $N_{\mathrm{tot}}$, which limits the noise floor, as shown in Figure 7, the atom temperature $T_{\mathrm{at}}$ and the effect related to it, such as the magnetic field gradient, should also be considered.

Due to the finite temperature of atoms [46,50,51], the expansion of the atom cloud and the magnetic field gradient are coupled to bring decoherence to the Ramsey interference, leading to a decrease in the fringe contrast. Because of the magnetic field gradient and the spatial distribution of the atoms in the atom cloud, each atom experiences a different variation of the magnetic field during the free fall.

We use the normal distribution as the distribution of position and momentum for atoms in an atom cloud. During the free fall, we calculate the atom trajectory and, thus, the magnetic field variation and then calculate the probability of detection for each atom. The outcome of the interference is the average of each atom’s probability. Details of the calculation are presented in Appendix B.

**Figure 8:**
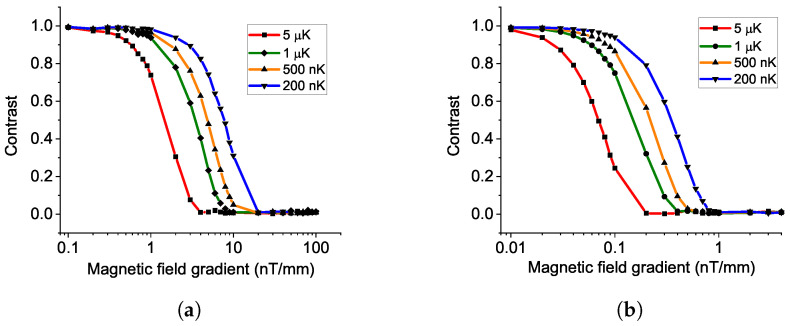
Decrease in fringe contrast by magnetic field gradient and atom temperature in free falling condition, with an evolution time of (**a**) 21ms and (**b**) 100ms.

From the result in Figure 8a, setting the free-falling time as 21ms, with an atom temperature of 500nK, to keep the contrast higher than 50%, the magnetic field gradient is required to be less than 5nT/mm. The colder the atom temperature, the higher the tolerance to the B field gradient. This effect is more obvious for higher free falling time. In Figure 8b, for an atom temperature of 500nK, with free falling time of 100ms, the B field gradient is required to be lower than 0.2nT/nm. In a metal vacuum chamber, with a conventional cooling technique such as optical molasses reaching 1 μK, a magnetic field gradient such as 10nT/mm [52] can wash out the interference fringe for free falling time, reaching the level of 10ms.

Experimentally, a scan for the magnetic field along the atomic trajectory is helpful in probing the homogeneity of the magnetic field inside atom interference devices [39,52].

There is a trade-off between atom number and atom temperature in the evaporation cooling technique. Meanwhile, the quantum projection noise affected by the atom number limits the noise floor. The atom temperature and the magnetic field gradient are coupled to limit the fringe contrast. With overall consideration of designing an experiment aiming for the one-shot resolution of 1pT, we show the recommended experimental conditions in Table 1.

The required atom number and temperature are conventional for results that achieved evaporation cooling producing BEC [46,53,54]. Still, there is plenty of room compared to the world’s top-level result [55]. The requirement on the B field gradient can be verified by the method from Refs. [39,52].

## 5. Noise Estimation

In this section, we present noise analysis in the proposed multi-wave atom interference magnetometer with the demonstration of a sensitivity function in a three-wave system and the resulting noise budget list.

### 5.1. Sensitivity Function in Three-Wave System

**Figure 9:**
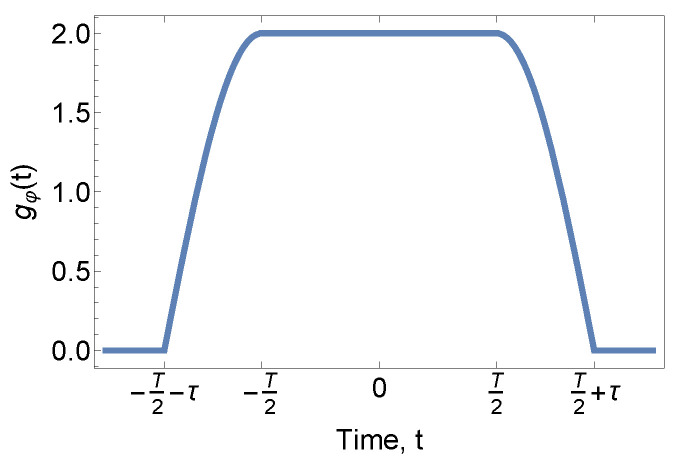
Sensitivity function for RF phase noise.

We deduce the sensitivity functions to the two most common noises affecting the measurement resolution, the phase noise of RF and the ambient high-frequency magnetic field noise. According to Ref. [43], the sensitivity function to phase noise is defined as Equation (8). Following the calculation sequence in Ref. [56] to insert phase noise $\varphi_{\mathrm{RF}}+\delta \varphi$ in the quantum state of each step in the Ramsey sequence, as listed from Equation (A15) to Equation (A17), $g_{\varphi }\left(t\right)$ is shown as Equation (9) and is sketched in Figure 9.

(8) $$g_{\varphi }\left(t\right)=2\lim_{\delta \varphi \to 0}\frac{\delta P(\delta \varphi ,t)}{\delta \varphi }$$

(9) $$g_{\varphi }\left(t\right)=\left\{\begin{array}{cc} 2\cos\left(\Omega_{\mathrm{eff}}\left(t+\frac{T}{2}\right)\right) & -\frac{T}{2}-\tau \leq t<-\frac{T}{2}\\ 2 & -\frac{T}{2}\leq t\leq \frac{T}{2}\\ 2\cos\left(\Omega_{\mathrm{eff}}\left(t-\frac{T}{2}\right)\right) & \frac{T}{2}<t\leq \frac{T}{2}+\tau \end{array}\right.$$

The sensitivity function for phase noise in Figure 9 is two times that in a typical two-level system [43] because the phase evolution is doubled, as in Equation (3). This maintains consistency with the fringe slope result in the previous section. Equation (9) is the exact phase noise sensitivity function in the F=1 system. For the F=2 system, the resulting sensitivity function can be approximated by scaling the fringe slope or can be calculated from scratch with Equation (8).

The transfer function of RF phase noise $H_{\varphi }\left(\omega \right)$, Equation (A23), can be obtained from 2πf times the Fourier transform of the sensitivity function $G_{\varphi }\left(\omega \right)$, Equation (A22). According to the relationship between phase noise and the magnetic field noise as in Equation (10) from Ref. [57], the transfer function of the magnetic field noise $H_{B}\left(\omega \right)$ is Equation (A24).

(10) $$H_{B}\left(\omega \right)=\kappa G_{\varphi }\left(\omega \right)$$

The phase uncertainty σ due to noise can be evaluated by

(11) $$\sigma^{2}=\int_{0}^{\infty }{\left|H\left(\omega \right)\right|}^{2}S\left(f\right)df.$$

Aiming at a resolution goal of 1pT with sensitivity as high as possible, supposing the noise spectrum S(f) as white noise from 0.1Hz to 100kHz, by applying Equation (11), the phase noise $S_{\varphi }\left(f\right)$ is required to reach $-114.6\;{\mathrm{dBrad}}^{2}/\mathrm{Hz}$ and the magnetic field noise $S_{B}\left(f\right)$ is required to reach $-30.4\;{\mathrm{dBpT}}^{2}/\mathrm{Hz}$.

### 5.2. Noise Budget

In addition to the RF phase noise or magnetic field noise, [45] the detection noise $\sigma_{P(\mathrm{DN})}$ coming from the detection beams and the noise when employing Raman transition $\sigma_{P(\mathrm{RAMAN})}$ at the final step in Figure 1, for example, should also be considered. These types of noise act as $\sigma_{P}$ in Equation (7) to contribute to the overall statistical noise.

In Table 2, the requirement for RF phase noise is conventional according to Ref. [58]. For the requirement of magnetic field noise, there is room compared to the ambient magnetic field noise, roughly $1\;\mathrm{fT}/\sqrt{\mathrm{Hz}}$ [21]. The detection noise is demonstrated in Ref. [45]. Table 2 estimates the noise-limited resolution per shot. The cycle time $T_{c}$, including the duration for MOT-loading and atom evaporation is of the order of 1−10s, as shown in Ref. [46]. The estimated resolution at 1s is thus multiplied by $\sqrt{T_{c}}$. The shot-by-shot operation mode, together with the need for a long integration time to lower the statistical noise if necessary, limits the expected sensitivity and the frequency range of the measurement to DC or near DC.

## 6. Discussion

### 6.1. Validity of the Model

When choosing RF pulses to form the magnetometer, the contribution of the oscillating RF field to the total magnetic field must be considered. Projected to the quantization axis, $B_{0}$ can be decomposed to $B_{⊥}$ and $B_{z}$, respectively, perpendicular and parallel to the quantization axis. By analogy, with the AC stark shift from an oscillating electric field, an oscillating magnetic field causes an energy shift. The energy shift caused by $B_{⊥}$ and $B_{z}$ can be measured according to Ref. [59].

The calculation is based on the RWA approximation, supposing $\delta_{R}\ll \kappa B$. The condition to the RWA approximation is the weak coupling condition $\Omega_{R}\ll \kappa B$ [60]. Together with the strong driving condition $\delta_{R}\ll \Omega_{R}$, the validity of the analytical results is under the overall condition $\delta_{R}\ll \Omega_{R}\ll \kappa B$. The real constraint is between $\Omega_{R}$ and the amplitude of *B*, especially close to or below the level of 1mG. As reported in Ref. [61], to measure the magnetic field of ∼1mG, a single ∼350Hz RF pulse of 100ms duration reveals a Rabi frequency in the order of 2π×5Hz. For the calculation, one can always set $\delta_{R}$ near zero and a low Rabi frequency to meet the overall condition. However, such a low Rabi frequency is not considered experimentally common because the longer the pulse duration, the more sources of decoherence kick in. The strong-coupling regime where $\Omega_{R}\gg \kappa B$ is out of the scope of the article.

### 6.2. Systematic Bias Estimation

In our modeling and demonstrated calculation, only the first-order Zeeman effect is considered. Taking into account the second-order Zeeman effect, together with the difference of first-order Zeeman coefficients between the system of F=1 and of F=2, different phase shifts in each channel of the dual atom-magnetometer will be measured as systematic bias. The more detailed Zeeman shifts [62] $E_{F}$ are given by

(12) $$E_{F=1}/\hbar =\kappa_{1}B-3\beta B^{2}$$

and

(13) $$E_{F=2}/\hbar =\kappa_{2}B+3\beta B^{2},$$

where $\kappa_{1}=2\pi \times 702.37\;\mathrm{kHz}/G$, $\kappa_{2}=2\pi \times 699.58\;\mathrm{kHz}/G$, and $\beta =2\pi \times 71.89\;\mathrm{Hz}/G^{2}$. When performing measurements using our dual magnetometer proposal, and without spatial separation of atom wave packets during initial state preparation, the observed different fringe shifts reveal the difference of Zeeman effect coefficients.

### 6.3. Trapped Configuration

The proposed magnetometer and the noise analysis are demonstrated with the free-falling configuration. Experiments can be conducted in an optical dipole trap or in a zero-gravity environment to increase the *B* variance tolerance. It should be noted that in an optical dipole trap, spin-dependent interactions [63,64,65] should be considered. In a magnetic trap, atoms only stay in the trap with low-field-seeker states. Forced use of the RF Ramsey sequence results in atom loss [41], leading to a fast reduction in fringe contrast. The quality of the state preparation should also be considered. Similar to the state preparation in atom clocks [66], atoms in states other than $m_{F}=0$ cause a reduction in fringe contrast. A proper optical pumping scheme [67] without significant atom losses is suggested. Spin-dependent interactions, together with the stability of the ambient magnetic field and temperature of atoms, bring a challenge to the long coherence time.

### 6.4. Quasi-Non-Destructive Detection

Excluding the non-resonant phase-contrast imaging method [68], just by employing the most common resonant absorption imaging method, in our proposed sequence, after detecting the atoms in F=2 system, a microwave or Raman pulse transfers atoms in the |1,0〉 state to the |2,0〉 state for absorption detection. Therefore, only the |1,0〉 state is destroyed. The remained quantum state is able to provide the quantum superposition facility without the necessity for one more instance of state preparation. An extended free evolution time and a readout pulse can reveal the fringe pattern for further steps. This brings the possibility of a continuous quasi-non-destructive experimental study of magnetic field effects in one shot, increasing the detection efficiency.

## 7. Conclusions

We have presented the proposal for a novel quantum magnetometer based on atoms’ multi-wave Ramsey interference, coupled by RF radiation. We have demonstrated the ***applicable interference fringes,*** which are essential for the next step of precision magnetic field measurements. Assuming a target resolution of 1pT, we have demonstrated reasonable ***experimental conditions,*** including atom status and the effect of the magnetic field gradient. The sensitivity functions in a multi-wave system are deduced to estimate the ***noise budget*** for the target resolution. The validity of the model and the trapped configuration are also discussed. Moreover, the dual-channel configuration features bias estimation and state detection with high efficiency. The dual-channel design makes it possible to adjust the ratio of atoms in each channel, meeting the different experimental requirements between sensitivity and functionality. As an alternative method for magnetic field sensing, especially in DC and low-frequency range, the proposal can be compared to the existing magnetometers to extend the study in the domain of magnetometry and atom interferometry.

## Figures and Tables

**Table 1:** Conditions required aiming for a magnetic field measurement one-shot resolution of 1pT.

Experimental Conditions	
Evolution time	≥21ms
Atom number	≥$1\times 10^{5}$
Atom temperature	≤500nK
B field gradient for 50% contrast	≤4nT/mm

**Table 2:** List of noise sources and their estimated effect on one-shot resolution.

Noise Source	Level	$\sigma_{B}$ (pT)
RF phase noise	$-114.6\;{\mathrm{dBrad}}^{2}/\mathrm{Hz}$	0.5
Magnetic field noise	$-30.4\;{\mathrm{dBpT}}^{2}/\mathrm{Hz}$	0.5
Quantum projection noise	$1.6\times 10^{-3}$	1.0
Detection noise	$1.8\times 10^{-3}$	1.2
Raman detection noise	$1.3\times 10^{-3}$	0.8
Total noise	1.9pT	

**Figure A2:**
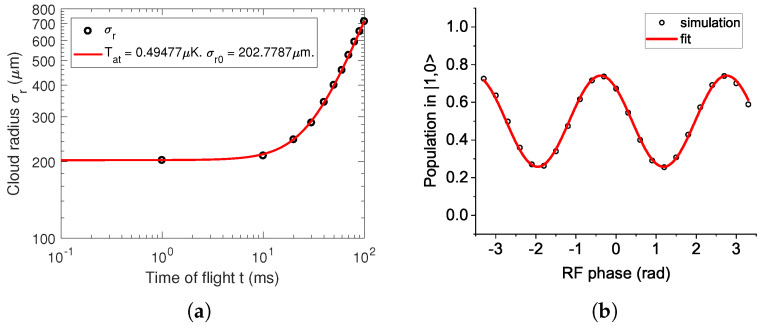
(**a**) Simulation of expansion of atom cloud during free fall, the red fitted line shows the atom temperature of 500nK and initial cloud radius of 200μm. (**b**) Simulation of decay of fringe contrast for a thermal atom cloud employed by Ramsey sequence of two π/2 pulses.

## Data Availability

Codes and data are available upon reasonable requests from the authors.

## Appendix A. Rabi Oscillation

The evolution of the system is the solution to the rotating-wave-approximation (RWA) time-dependent Schrödinger equation

(A1) $$i\hbar \dot{c}=Hc,$$

where c is the N-level state vector, respectively, $c_{1}={\left(c_{11},c_{12},c_{13}\right)}^{T}$ containing the amplitudes for $|F=1,m_{F}=-1\rangle$, |1,0〉, |1,+1〉 spin states, and $c_{2}={\left(c_{21},c_{22},c_{23},c_{24},c_{25}\right)}^{T}$ containing the amplitudes for |2,−2〉, |2,−1〉, |2,0〉, |2,+1〉, |2,+2〉 spin states.

The Hamiltonian for a three-level atom-photon system is demonstrated in Ref. [34]. The one for a five-level system is demonstrated in Ref. [26]. Taking Ref. [48] as the reference, in this paper, the Hamiltonians to describe the multilevel system of ${}^{87}$Rb $5^{2}S_{1/2}$ are Equation (A2) for F=1 and Equation (A3) for F=2, where $\delta_{R}=\omega_{\mathrm{RF}}-\kappa B$, $\kappa =\mu_{B}/\left(2\hbar \right)$, $\mu_{B}$ is the Bohr magneton and $\Omega_{R}$ is the Rabi frequency quantifying the coupling strength.

(A2) $$H_{R1}=\hbar \left(\begin{array}{ccc} -\delta_{R} & \sqrt{2}\Omega_{R}e^{i\varphi_{\mathrm{RF}}} & 0\\ \sqrt{2}\Omega_{R}e^{-i\varphi_{\mathrm{RF}}} & 0 & \sqrt{2}\Omega_{R}e^{i\varphi_{\mathrm{RF}}}\\ 0 & \sqrt{2}\Omega_{R}e^{-i\varphi_{\mathrm{RF}}} & \delta_{R} \end{array}\right)$$

(A3) $$H_{R2}=\hbar \left(\begin{array}{ccccc} 2\delta_{R} & 2\Omega_{R}e^{i\varphi_{\mathrm{RF}}} & 0 & 0 & 0\\ 2\Omega_{R}e^{-i\varphi_{\mathrm{RF}}} & \delta_{R} & \sqrt{6}\Omega_{R}e^{i\varphi_{\mathrm{RF}}} & 0 & 0\\ 0 & \sqrt{6}\Omega_{R}e^{-i\varphi_{\mathrm{RF}}} & 0 & \sqrt{6}\Omega_{R}e^{i\varphi_{\mathrm{RF}}} & 0\\ 0 & 0 & \sqrt{6}\Omega_{R}e^{-i\varphi_{\mathrm{RF}}} & -\delta_{R} & 2\Omega_{R}e^{i\varphi_{\mathrm{RF}}}\\ 0 & 0 & 0 & 2\Omega_{R}e^{-i\varphi_{\mathrm{RF}}} & -2\delta_{R} \end{array}\right)$$

Different from Refs. [47,48], in the coupling terms of Equations (A2) and (A3), there is an additional phase factor $e^{i\varphi_{\mathrm{RF}}}$. We use this phase to insert phase jumps to perform noise analysis under the framework of the atom interferometer in Section 5. This phase is close to that in Ref. [26]. Equation (A2) is close to the result in Ref. [34], which contains an extra phase factor from the AC stark shift.

Under the strong driving condition, $\delta_{R}\ll \Omega_{R}$, after employing the techniques in Ref. [47], the time evolution transfer matrix of Equations (A2) and (A3) are Equations (A4) and (A5), where $\Omega_{\mathrm{eff}}=\sqrt{\delta_{R}^{2}+4\Omega_{R}^{2}}$.

(A4) $$U_{R1}\left(t\right)=\frac{1}{2}\left(\begin{array}{ccc} \cos\left(\Omega_{\mathrm{eff}}t\right)+1 & -\sqrt{2}ie^{i\varphi_{\mathrm{RF}}}\sin\left(\Omega_{\mathrm{eff}}t\right) & e^{2i\varphi_{\mathrm{RF}}}\left(\cos\left(\Omega_{\mathrm{eff}}t\right)-1\right)\\ -\sqrt{2}ie^{-i\varphi_{\mathrm{RF}}}\sin\left(\Omega_{\mathrm{eff}}t\right) & 2\cos\left(\Omega_{\mathrm{eff}}t\right) & -\sqrt{2}ie^{i\varphi_{\mathrm{RF}}}\sin\left(\Omega_{\mathrm{eff}}t\right)\\ e^{-2i\varphi_{\mathrm{RF}}}\left(\cos\left(\Omega_{\mathrm{eff}}t\right)-1\right) & -\sqrt{2}ie^{-i\varphi_{\mathrm{RF}}}\sin\left(\Omega_{\mathrm{eff}}t\right) & \cos\left(\Omega_{\mathrm{eff}}t\right)+1 \end{array}\right)$$

(A5) $$U_{R2}\left(t\right)=\frac{1}{2}\left(S_{a}S_{b}S_{c}S_{d}S_{e}\right),$$

where the column vectors S are

$$S_{a}=\left(\begin{array}{c} \frac{1}{2}\left(1+\cos(\Omega_{\mathrm{eff}}t)\right){}^{2}\\ -ie^{-i\varphi_{\mathrm{RF}}}\sin\left(\Omega_{\mathrm{eff}}t\right)\left(1+\cos(\Omega_{\mathrm{eff}}t)\right)\\ \frac{1}{2}\sqrt{\frac{3}{2}}e^{-2i\varphi_{\mathrm{RF}}}\left(\cos\left(2\Omega_{\mathrm{eff}}t\right)-1\right)\\ ie^{-3i\varphi_{\mathrm{RF}}}\sin\left(\Omega_{\mathrm{eff}}t\right)\left(1-\cos\left(\Omega_{\mathrm{eff}}t\right)\right)\\ \frac{1}{2}e^{-4i\varphi_{\mathrm{RF}}}\left(1-\cos\left(\Omega_{\mathrm{eff}}t\right)\right){}^{2} \end{array}\right),$$

$$S_{b}=\left(\begin{array}{c} -ie^{i\varphi_{\mathrm{RF}}}\sin\left(\Omega_{\mathrm{eff}}t\right)\left(1+\cos\left(\Omega_{\mathrm{eff}}t\right)\right)\\ \left(2\cos\left(\Omega_{\mathrm{eff}}t\right)-1\right)\left(\cos\left(\Omega_{\mathrm{eff}}t\right)+1\right)\\ -\sqrt{\frac{3}{2}}ie^{-i\varphi_{\mathrm{RF}}}\sin\left(2\Omega_{\mathrm{eff}}t\right)\\ e^{-2i\varphi_{\mathrm{RF}}}\left(2\cos\left(\Omega_{\mathrm{eff}}t\right)+1\right)\left(\cos\left(\Omega_{\mathrm{eff}}t\right)-1\right)\\ ie^{-3i\varphi_{\mathrm{RF}}}\sin\left(\Omega_{\mathrm{eff}}t\right)\left(1-\cos\left(\Omega_{\mathrm{eff}}t\right)\right) \end{array}\right),$$

$$S_{c}=\left(\begin{array}{c} \frac{1}{2}\sqrt{\frac{3}{2}}e^{2i\varphi_{\mathrm{RF}}}\left(\cos\left(2\Omega_{\mathrm{eff}}t\right)-1\right)\\ -\sqrt{\frac{3}{2}}ie^{i\varphi_{\mathrm{RF}}}\sin\left(2\Omega_{\mathrm{eff}}t\right)\\ \frac{1}{2}\left(3\cos\left(2\Omega_{\mathrm{eff}}t\right)+1\right)\\ -\sqrt{\frac{3}{2}}ie^{-i\varphi_{\mathrm{RF}}}\sin\left(2\Omega_{\mathrm{eff}}t\right)\\ \frac{1}{2}\sqrt{\frac{3}{2}}e^{-2i\varphi_{\mathrm{RF}}}\left(\cos\left(2\Omega_{\mathrm{eff}}t\right)-1\right) \end{array}\right),$$

$$S_{d}=\left(\begin{array}{c} ie^{3i\varphi_{\mathrm{RF}}}\sin\left(\Omega_{\mathrm{eff}}t\right)\left(1-\cos\left(\Omega_{\mathrm{eff}}t\right)\right)\\ e^{2i\varphi_{\mathrm{RF}}}\left(2\cos\left(\Omega_{\mathrm{eff}}t\right)+1\right)\left(\cos\left(\Omega_{\mathrm{eff}}t\right)-1\right)\\ -\sqrt{\frac{3}{2}}ie^{i\varphi_{\mathrm{RF}}}\sin\left(2\Omega_{\mathrm{eff}}t\right)\\ \left(2\cos\left(\Omega_{\mathrm{eff}}t\right)-1\right)\left(\cos\left(\Omega_{\mathrm{eff}}t\right)+1\right)\\ -ie^{-i\varphi_{\mathrm{RF}}}\sin\left(\Omega_{\mathrm{eff}}t\right)\left(1+\cos\left(\Omega_{\mathrm{eff}}t\right)\right) \end{array}\right),$$

$$S_{e}=\left(\begin{array}{c} \frac{1}{2}e^{4i\varphi_{\mathrm{RF}}}\left(1-\cos\left(\Omega_{\mathrm{eff}}t\right)\right){}^{2}\\ ie^{3i\varphi_{\mathrm{RF}}}\sin\left(\Omega_{\mathrm{eff}}t\right)\left(1-\cos\left(\Omega_{\mathrm{eff}}t\right)\right)\\ \frac{1}{2}\sqrt{\frac{3}{2}}e^{2i\varphi_{\mathrm{RF}}}\left(\cos\left(2\Omega_{\mathrm{eff}}t\right)-1\right)\\ -ie^{i\varphi_{\mathrm{RF}}}\sin\left(\Omega_{\mathrm{eff}}t\right)\left(1+\cos\left(\Omega_{\mathrm{eff}}t\right)\right)\\ \frac{1}{2}\left(1+\cos\left(\Omega_{\mathrm{eff}}t\right)\right){}^{2} \end{array}\right).$$

Due to the equality feature of trigonometric identities, different forms of Equations (A4) and (A5) exist. We present here the power-reduced forms, which easily reveal the state evolution periodicity.

Equations (A4) and (A5) describe the state of evolution when atoms are shined by RF radiation. Knowing the state at any time *t* as c(t), after radiation duration τ, the state c(t+τ) is

(A6) $$c\left(t+\tau \right)=U_{R}\left(\tau \right)c\left(t\right),$$

where $U_{R}$ can be $U_{R1}$ or $U_{R2}$. In this way, the Schrödinger differential equation becomes a one step matrix operation.

We evaluate the Rabi oscillation with different pulse duration τ. The population of atoms P(t) on each spin state can be obtained from the modulo square of each component of c(t). Experimentally, the initial quantum state can be easily prepared at a side state, the highest or the lowest $m_{F}$ state, or a center state, the $m_{F}=0$ state. Thus, we demonstrate these two situations. The Rabi oscillation in the F=1 system when the initial state is |1,−1〉 and |1,0〉 are Equations (A7) and (A8). Respectively, the Rabi oscillation for the F=2 system when the initial state is |2,−2〉 and |2,0〉 are Equations (A9) and (A10). We compare the results sketched in Figure A1.

With state initialization on |1,−1〉,

(A7) $$P{\left(\tau \right)}_{|F=1\rangle }=\frac{1}{4}\left(\begin{array}{c} \left(1+\cos\left(\Omega_{\mathrm{eff}}\tau \right)\right){}^{2}\\ 1-\cos\left(2\Omega_{\mathrm{eff}}\tau \right)\\ \left(1-\cos\left(\Omega_{\mathrm{eff}}\tau \right)\right){}^{2} \end{array}\right).$$

With state initialization on |1,0〉

(A8) $$P{\left(\tau \right)}_{|F=1\rangle }=\frac{1}{4}\left(\begin{array}{c} 1-\cos\left(2\Omega_{\mathrm{eff}}\tau \right)\\ 2\left(1+\cos\left(2\Omega_{\mathrm{eff}}\tau \right)\right)\\ 1-\cos\left(2\Omega_{\mathrm{eff}}\tau \right) \end{array}\right).$$

With state initialization on |2,−2〉

(A9) $$P{\left(\tau \right)}_{|F=2\rangle }=\frac{1}{32}\left(\begin{array}{c} 2\left(1+\cos\left(\Omega_{\mathrm{eff}}\tau \right)\right){}^{4}\\ 4\left(1+\cos\left(\Omega_{\mathrm{eff}}\tau \right)\right){}^{2}\left(1-\cos\left(2\Omega_{\mathrm{eff}}\tau \right)\right)\\ 3\left(1-\cos\left(2\Omega_{\mathrm{eff}}\tau \right)\right){}^{2}\\ 4\left(1-\cos\left(\Omega_{\mathrm{eff}}\tau \right)\right){}^{2}\left(1-\cos\left(2\Omega_{\mathrm{eff}}\tau \right)\right)\\ 2\left(1-\cos\left(\Omega_{\mathrm{eff}}\tau \right)\right){}^{4} \end{array}\right).$$

With state initialization on |2,0〉

(A10) $$P{\left(\tau \right)}_{|F=2\rangle }=\frac{1}{32}\left(\begin{array}{c} 3\left(1-\cos\left(2\Omega_{\mathrm{eff}}\tau \right)\right){}^{2}\\ 6\left(1-\cos\left(4\Omega_{\mathrm{eff}}\tau \right)\right)\\ 2\left(1+3\cos\left(2\Omega_{\mathrm{eff}}\tau \right)\right){}^{2}\\ 6\left(1-\cos\left(4\Omega_{\mathrm{eff}}\tau \right)\right)\\ 3\left(1-\cos\left(2\Omega_{\mathrm{eff}}\tau \right)\right){}^{2} \end{array}\right).$$

There are several features in Figure A1. First, not all the states are linear transformations of the sine function, especially in the hF=2 system. In the case of F=2, the eigenvalues of $H_{R2}$ are 0, $\pm \Omega_{\mathrm{eff}}$ and $\pm 2\Omega_{\mathrm{eff}}$. The evolution is complicated by the wave frequencies and high harmonics in wave functions of the different matter. Second, when the initial state is a side state, only the two opposite side states have a full population inversion. The pulse area $\Omega_{\mathrm{eff}}\tau$ needed for the inversion is π and one cycle is 2π. Meanwhile, although the center state does not reach a complete population inversion, the periodicity is two times as fast as that of the side states. Third, if and only if the atoms are prepared in the center state, their evolution frequency is doubled and a complete population inversion is kept. The pulse area for one cycle is π instead of 2π. The last feature is important for producing an interference fringe with full contrast and with a high fringe slope in an atom interferometer. The fringe slopes in the next section prove this feature. The highest fringe slope can be obtained when preparing an initial center state.

The initial RF phase $\varphi_{\mathrm{RF}}$ is set to be time-independent in this section; therefore, $\varphi_{\mathrm{RF}}$ only exists in transfer matrices Equations (A4) and (A5). It vanishes after the operation of the modulo square to obtain the Rabi oscillations as Equations (A7)–(A10). The results are consistent with Refs. [29,35].

(A11) $$U_{R1f}\left(t\right)=\left(\begin{array}{ccc} e^{i\delta_{R}t} & 0 & 0\\ 0 & 1 & 0\\ 0 & 0 & e^{-i\delta_{R}t} \end{array}\right)$$

(A12) $$U_{R2f}\left(t\right)=\left(\begin{array}{ccccc} e^{-i2\delta_{R}t} & 0 & 0 & 0 & 0\\ 0 & e^{-i\delta_{R}t} & 0 & 0 & 0\\ 0 & 0 & 1 & 0 & 0\\ 0 & 0 & 0 & e^{i\delta_{R}t} & 0\\ 0 & 0 & 0 & 0 & e^{i2\delta_{R}t} \end{array}\right)$$

## Appendix B. Simulation of Fringe Contrast Decay

We use the normal distribution as the distribution of position and momentum for atoms in a thermal atom cloud. At time t=0, the standard deviation in position space $\sigma_{r}$ is related to the initial radius of the atoms $r_{\mathrm{at}0}$, and the standard deviation in momentum space is related to the atom temperature $T_{\mathrm{at}}$ as $\sigma_{v}^{2}=\sqrt{k_{B}T_{\mathrm{at}}/m_{\mathrm{Rb}}}$. During the free fall, due to the distribution in momentum space, the distribution in position space is expanding, as shown in Figure A2a, leading to the expansion of the atom cloud, which can be characterized by Equation (A13). The magnetic field felt by each atom during the free fall can be calculated by the position and the field gradient. The time sequence is divided by finite elements with a duration dt of 10μs.

(A13) $$\sigma_{r}^{2}\left(t\right)=r_{\mathrm{at}0}^{2}+\frac{k_{B}T_{\mathrm{at}}}{m_{\mathrm{Rb}}}t^{2}$$

After repeating the Ramsey sequence calculation for each atom, as we demonstrated in Section 3, we obtain the resulting probability as the mean value of the probability of each atom. By scanning the RF phase, the decay of the fringe contrast is observed by the simulated fringe, as shown in Figure A2b. We set the bias magnetic field as 50mG, and its direction is along the gravity. The assumed magnetic field gradient in three dimensions is uniform.

## Appendix C. Calculation of Sensitivity Function in Section 5

The details of the calculation of phase noise sensitivity function $g_{\varphi }\left(t\right)$ in a three-level system are listed below.

In a Ramsey sequence consisting of two pulses with pulse area $\Omega_{\mathrm{eff}}\tau$ of π/2 and a free evolution time *T* centered at time t=0, the three time periods are from $-\frac{T}{2}-\tau$ to $-\frac{T}{2}$, from $-\frac{T}{2}$ to $\frac{T}{2}$, and from $\frac{T}{2}$ to $\frac{T}{2}+\tau$. We determine $g_{\varphi }\left(t\right)$ in the F=1 system, with state initialization on |1,0〉, at the output point $P_{R1(\pi /2)}=\frac{1}{2}$ on Ramsey fringe of the |1,0〉 state, as shown in Figure 4a. The $P=\frac{1}{2}$ output point is achieved by setting $\delta_{R}=0$ in the pulse steps and $\delta_{R}T=\frac{\pi }{4}$ during the free evolution time. The RF phase noise is defined as a phase step from 0 to δφ at time *t* during the Ramsey sequence.

To clarify the notation in the following equations, we reform Equation (A4) to Equation (A14).

(A14) $$U_{R1}\left(t,\varphi \right)=\frac{1}{2}\left(\begin{array}{ccc} \cos\left(\Omega_{\mathrm{eff}}t\right)+1 & -\sqrt{2}ie^{i\varphi }\sin\left(\Omega_{\mathrm{eff}}t\right) & e^{2i\varphi }\left(\cos\left(\Omega_{\mathrm{eff}}t\right)-1\right)\\ -\sqrt{2}ie^{-i\varphi }\sin\left(\Omega_{\mathrm{eff}}t\right) & 2\cos\left(\Omega_{\mathrm{eff}}t\right) & -\sqrt{2}ie^{i\varphi }\sin\left(\Omega_{\mathrm{eff}}t\right)\\ e^{-2i\varphi }\left(\cos\left(\Omega_{\mathrm{eff}}t\right)-1\right) & -\sqrt{2}ie^{-i\varphi }\sin\left(\Omega_{\mathrm{eff}}t\right) & \cos\left(\Omega_{\mathrm{eff}}t\right)+1 \end{array}\right)$$

(A15) $$c\left(\frac{T}{2}+\tau \right)=U_{R1}\left(\tau ,\delta \varphi \right)U_{R1f}\left(T\right)U_{R1}\left(-\frac{T}{2}-t,\delta \varphi \right)U_{R1}\left(t-\left(-\frac{T}{2}-\tau \right),0\right)c\left(-\frac{T}{2}-\tau \right)$$

(A16) $$\begin{array}{cc} P_{|1,0\rangle }= & \frac{1}{32}(2\cos\left(\delta \varphi \right)-2\cos\left(\Omega_{\mathrm{eff}}\left(2t+T\right)\right)+\cos\left(\delta \varphi +\Omega_{\mathrm{eff}}\left(2t+T\right)\right)+\cos\left(\delta \varphi -\Omega_{\mathrm{eff}}\left(2t+T\right)\right)\\ \; & +2\left(1+\sin\left(\delta \varphi +\frac{1}{2}\Omega_{\mathrm{eff}}\left(2t+T\right)\right)+\sin\left(\delta \varphi -\frac{1}{2}\Omega_{\mathrm{eff}}\left(2t+T\right)\right)\right){)}^{2} \end{array}$$

(A17) $$c\left(\frac{T}{2}+\tau \right)=U_{R1}\left(\frac{T}{2}+\tau -t,\delta \varphi \right)U_{R1}\left(t-\frac{T}{2},0\right)U_{R1f}\left(T\right)U_{R1}\left(\tau ,0\right)c\left(-\frac{T}{2}-\tau \right)$$

(A18) $$\begin{array}{cc} P_{|1,0\rangle }= & \frac{1}{8}(\cos\left(\delta \varphi \right)\left(1+\cos\left(\Omega_{\mathrm{eff}}\left(2t-T\right)\right)\right)\\ \; & +2\left(\sin\left(\delta \varphi \right)\cos\left(\Omega_{\mathrm{eff}}\left(t-\frac{T}{2}\right)\right)+\sin^{2}\left(\Omega_{\mathrm{eff}}\left(t-\frac{T}{2}\right)\right)\right){)}^{2} \end{array}$$

Under these conditions, when the phase step occurs during the first pulse, from $-\frac{T}{2}-\tau$ to $-\frac{T}{2}$, the final state $c(\frac{T}{2}+\tau )$ is calculated as Equation (A15). The probability at |1,0〉 output is Equation (A16).

According to the definition of Equation (8), the resulting phase noise sensitivity function with noise inserted during the first pulse is Equation (A19).

(A19) $$g_{\varphi }\left(t\right)=2\lim_{\delta \varphi \to 0}\frac{P_{|1,0\rangle }-\frac{1}{2}}{\delta \varphi }=2\cos\left(\Omega_{\mathrm{eff}}\left(t+\frac{T}{2}\right)\right)$$

The final state with phase noise inserted during the free evolution is Equation (A20), and the calculated probability is Equation (A21). The phase step that occurred at any time *t* during the free evolution is considered a phase shift at the start of the second pulse. For this physics reason, there is no step split caused by inserting the phase noise into Equation (A20).

(A20) $$c\left(\frac{T}{2}+\tau \right)=U_{R1}\left(\tau ,\delta \varphi \right)U_{R1f}\left(T\right)U_{R1}\left(\tau ,0\right)c\left(-\frac{T}{2}-\tau \right)$$

(A21) $$P_{|1,0\rangle }=\frac{1}{2}\left(1+\sin\left(2\delta \varphi \right)\right)$$

The final state with phase noise inserted during the second pulse is Equation (A17), and the calculated probability is Equation (A18).

The entire form of the phase noise sensitivity function is arranged as Equation (9).

(A22) $${\left|G_{\varphi }\left(\omega \right)\right|}^{2}=\frac{16\Omega_{\mathrm{eff}}^{2}{\left(\omega \cos\left(\left(\frac{T}{2}+\tau \right)\omega \right)+\Omega_{\mathrm{eff}}\sin\left(\frac{T}{2}\omega \right)\right)}^{2}}{\omega^{2}{\left(\omega^{2}-\Omega_{\mathrm{eff}}^{2}\right)}^{2}}$$

(A23) $${\left|H_{\varphi }\left(\omega \right)\right|}^{2}=\frac{16\Omega_{\mathrm{eff}}^{2}{\left(\omega \cos\left(\left(\frac{T}{2}+\tau \right)\omega \right)+\Omega_{\mathrm{eff}}\sin\left(\frac{T}{2}\omega \right)\right)}^{2}}{{\left(\omega^{2}-\Omega_{\mathrm{eff}}^{2}\right)}^{2}}$$

(A24) $${\left|H_{B}\left(\omega \right)\right|}^{2}=\frac{16\kappa^{2}\Omega_{\mathrm{eff}}^{2}{\left(\omega \cos\left(\left(\frac{T}{2}+\tau \right)\omega \right)+\Omega_{\mathrm{eff}}\sin\left(\frac{T}{2}\omega \right)\right)}^{2}}{\omega^{2}{\left(\omega^{2}-\Omega_{\mathrm{eff}}^{2}\right)}^{2}}$$
